# From Isolation to Information: Launching an Online Community for Patients with Primary Sclerosing Cholangitis, Primary Biliary Cholangitis, and Autoimmune Hepatitis in Romania—A Descriptive Study

**DOI:** 10.3390/healthcare13233148

**Published:** 2025-12-02

**Authors:** Matei Mandea, Speranta M. Iacob, Maria Mandea, Mihaela C. Ghioca, Liliana S. Gheorghe

**Affiliations:** 1 Department of Internal Medicine, Discipline of Gastroenterology and Hepatology, University of Medicine and Pharmacy Carol Davila, 050474 Bucharest, Romania; msiacob@gmail.com (S.M.I.);; 2 Digestive Diseases and Liver Transplant Center, Fundeni Clinical Institute, 022328 Bucharest, Romania; 3 Department of Animation and Interactivity, Art of Game Design Master, National University of Theatral Art and Cinematography, 021452 Bucharest, Romania; maria.mandea@unatc.ro

**Keywords:** autoimmune liver diseases, primary sclerosing cholangitis, primary biliary cholangitis, quality of life, health literacy, online platform

## Abstract

**Background:** Primary Sclerosing Cholangitis (PSC), Primary Biliary Cholangitis (PBC), and Autoimmune Hepatitis (AIH) are rare immune-mediated liver conditions that significantly affect patients’ quality of life. In Romania, access to specialized information and patient support resources is limited, underscoring the need for tailored educational tools. The aim was to describe the methodology for developing, implementing, and conducting a feasibility study of an online platform for patients with PSC, PBC, and AIH, as a pilot study, providing early insights. **Methods:** The platform offers educational materials, registration, a discussion forum, and digital tools for quality-of-life assessment. Data on demographics, usage, and quality of life were collected using standardized questionnaires (CLDQ-PSC, PBC-10) and non-standardized questionnaires, and analyzed with Microsoft Office Excel and DATATab. **Results:** The website was created using an online platform requiring no advanced IT skills. Content was developed in accordance with international guidelines (EASL, AASLD) and translated and adapted for Romanian patients. As of 15 July 2025, 81 patients had been registered (26% PSC, 68% PBC, 6% AIH), with a predominance of urban participants (all patients: 87% female, mean age at diagnosis = 44.5 years). Most participants used mobile devices and reported improved understanding and engagement with their health after using the platform. **Conclusions:** The first dedicated digital platform has been established in Romania to address the health literacy needs of patients with PSC, PBC, and AIH. The study offers insights into future directions and a replicable model for similar initiatives. The pilot evaluation of the platform faced several limitations, including self-selection bias, non-standardized assessments, and a small sample size.

## 1. Introduction

Primary Sclerosing Cholangitis (PSC) and Primary Biliary Cholangitis (PBC) are two immune-mediated cholestatic liver diseases. Both are characterized by chronic inflammation of the bile ducts, leading to cholestasis either through inflammatory mechanisms or due to the development of bile duct stenosis. Although the pathophysiological mechanisms of these diseases differ, they share common symptoms, including pruritus, fatigue, abdominal pain, and jaundice [1,2,3,4,5]. PSC and PBC can be associated with autoimmune hepatitis (AIH). AIH can occur alone or with PSC and PBC [6,7]. It has a prevalence of 10.7–23.9 cases per 100,000 people in Europe and an incidence of 1.37 per 100,000 [6]. PSC, PBC, and AIH progress gradually, leading to liver fibrosis and cirrhosis, along with their complications such as encephalopathy, ascites, and variceal bleeding. Additionally, they increase the risk of bone metabolic disease [8,9,10]. The epidemiological data on these diseases in Romania are consistent; however, information from Europe indicates that PSC has a prevalence of 16 cases per 100,000 people, higher in men, and an incidence of 0.68–1.5 cases per 100,000 people. PBC has a prevalence of 22–35 cases and an incidence of 0.87–2.96 cases, being more common in women. The values are higher in countries in the northern part of the European continent and in the United States [11,12,13,14,15].

Information on health literacy in Romania is limited, especially regarding liver diseases. Available data come from oncology, infectious diseases, and dentistry [16,17,18,19]. Individuals with lower health literacy often face poorer health outcomes because they struggle to manage their conditions and utilize preventive and dietary measures. It can also cause psychosocial issues and raise healthcare costs [17,20,21,22,23].

These diseases progress over about 20 years before requiring liver transplant or causing death. Their rarity (fewer than 40 per 100,000) limits awareness of diagnosis and treatment, underscoring the need for better information sharing [10,24].

### 1.1. Health Literacy

Health literacy is the ability to understand and use basic health information for personal decisions, as defined by the WHO. It encompasses digital literacy, enabling individuals to assess online medical information [20,21,25]. Good health literacy improves quality of life by enhancing clinical outcomes, treatment adherence, and reducing costs [26]. The Health Belief Model (HBM) explains how perceptions of susceptibility, severity, benefits, and barriers influence health behaviors, such as screening and lifestyle changes in liver diseases [27,28]. Studies show low health literacy correlates with higher healthcare costs and poorer health outcomes in liver cirrhosis [29]. The Nutbeam model categorizes health literacy into three levels: functional, interactive, and critical, emphasizing both social and cognitive skills [30]. The PRECEDE-PROCEED Model (PPM) supports health interventions through diagnosis and evaluation, used in liver cancer to improve patient knowledge and behaviors [31]. Social Cognitive Therapy (SCT) links behavior to environment, guiding hepatitis C treatments by influencing social networks [32]. The Chronic Care Model (CCM) manages chronic diseases by fostering self-management skills. It has been applied to rare liver conditions like PSC and PBC, assessing patient perceptions and online health information-seeking behaviors. Overall, these models highlight the importance of health literacy in managing liver diseases and designing effective interventions [11].

### 1.2. Literacy Assessment

Health Literacy assessment can be conducted using questionnaires administered before and after providing the information to determine the usefulness and amount of information the educational tool offers [33]. These questionnaires can be non-standardized by asking questions about the level of education, knowledge of the disease, diagnosis, treatment, monitoring, and information sources [34,35].

Health Literacy assessment can be carried out using digital scales like the Mobile-Centered Digital Health Readiness—Health Literacy and Equity Scale (mDiHERS), which measures patients’ digital health skills, literacy, and access. Questionnaires can also be used before and after to evaluate the usefulness of the educational tool [34,35,36]. The eHealth Literacy Scale (eHEALS) assesses digital literacy and was used in Kelly et al.’s study on patients with chronic liver disease. It helps identify ways to improve digital literacy by evaluating the use of digital devices and the quality of information [37].

### 1.3. Online Platforms and Quality of Life Assessment

Multi-use online platforms integrate tools to provide patients with up-to-date information, enable registration, connect with health services, and measure QOL. In PSC or PBC, diseases impact daily life more than disease progression, as both are long-term conditions [38]. Current treatments less effectively reduce biochemical cholestasis. Consequently, patients face significant psychosocial challenges, including stigma, isolation, limited disease knowledge, and restricted access to specialized care. The key tools for monitoring QOL are Patient-Reported Outcomes (PROs) and generic questionnaires. Specific questionnaires have also been developed, such as the CLDQ-PSC (Chronic Liver Disease Questionnaire—Primary Sclerosing Cholangitis), PSC-PRO, and PBC-40 (with its short form—PBC-10) questionnaires [39,40,41,42,43,44]. These have a variable number of questions and require time to understand and complete.

Health literacy in rare liver diseases refers to the ability to access and understand the health information and services required in the field, thereby enabling better disease management and improved QOL [21,37]. In rare liver diseases, skills like critical analysis, functional literacy, managing uncertainty, and health literacy for discussing with healthcare providers are important [20,21,30,45]. In rare liver diseases, long-term adherence to treatment and investigations prevents complications. Nourian et al. demonstrated that monitoring, lifestyle changes, and education resulted in improved outcomes [27]. The greatest impact on QOL (QoL) across all three diseases is fatigue, as Michel et al. point out, followed by anxiety related to treatment uncertainty and long-term progression [46]. However, van Munster et al. note that this can be reduced through improvements in medical knowledge [21]. A study by Wunsch et al. demonstrated that confidence in treatment is a significant factor contributing to improved health-related quality of life (HRQOL) [38]. Access to health literacy and information about health services is more challenging. Guifarro et al. in Central America found that, although patients with autoimmune liver diseases like PSC wanted to join clinical trials, only 26% were asked [47]. As Kardashian et al. point out, there are gender-based and ethnic-based barriers to treatment access in chronic liver disease in the U.S., leading to a higher risk of complications and mortality [48].

### 1.4. Significance of an Online Platform

Digital health literacy in autoimmune liver diseases is a challenge in Romania due to limited information on disease epidemiology and patients’ access to health data [16,17,18,21,37,49]. Research on chronic liver disease shows a shortage of digital educational resources, exposing a gap between general digital knowledge and health-specific info. It also highlights limited research on autoimmune liver diseases, lacking epidemiological data, and emphasizes the high need for info on patients’ quality of life with PSC, PBC, and AIH [50]. There is also no data on how patients respond to available treatments. It introduces a way for patients to access information more easily, thereby improving health literacy [51]. It proposes a method for healthcare providers to communicate better with patients and collect vital data on quality of life, treatment adherence, and monitoring. Data gathered over time through questionnaires and symptom tracking will assist in understanding the disease’s natural history in Romania [37,52,53]. This will provide policymakers with valuable insights to enhance access to specialized care and approve and reimburse new treatments [48]. Health literacy correlates with poorer clinical outcomes and higher hospital costs, as noted by van Munster et al. [21]. A study showed that treatment adherence in patients with cirrhosis improves with higher health literacy.

The purpose of this paper is to detail the development process and a pilot assessment of the platform’s usefulness, and it also outlines potential future enhancements and directions for ongoing development. In this paper, we present a comprehensive overview of an online patient-facing platform, aligned with guidelines, designed to support Romanian patients diagnosed with PBC, PSC, or AIH. We examine its development, emphasize its practical benefits and accessibility for patients, and offer insights into prospects and its potential as a replicable model.

## 2. Materials and Methods

In this article, we presented the methodology for creating and implementing the online platform dedicated to health literacy, including the information provided, the utilities created, and its motivation. We conducted a descriptive observational pilot study with pre- and post-surveys to evaluate the online platform. The purpose of this paper was to describe the methodology for developing, implementing, and conducting a feasibility study of an online platform. The evaluation was not conducted in a standardized manner, utilizing validated tools for health literacy evaluation.

### 2.1. The Development Process of the Online Platform

The platform can be accessed at https://www.colangite.ro and is intended exclusively for Romanian-speaking patients, whether residing in Romania or abroad.

The website was created on an online platform that does not necessitate advanced IT knowledge or programming skills. In this way, the evolving knowledge about PSC, PBC, and AIH can be easily kept up to date by medical professionals responsible for the platform, a necessity as new research and treatment options for these diseases are constantly emerging.

The website was created after researching other websites dedicated to patients with PSC or PBC, specifically in terms of content, registration, and usage. We included in the analysis the platforms PSC-Partners, UK-PBC, ALBI-France, UK-PSC, and PBCers.

To present information on the three diseases, the EASL, AASLD, and ACG guidelines on PSC, PBC, and AIH were consulted, along with information on the liver’s anatomy and physiology. These sources were searched on PubMed and then translated into Romanian and adapted into non-medical language.

### 2.2. Evaluation of the Platform

This study employed a general, non-standardized questionnaire on platform use, the CLDQ-PSC questionnaire to assess the QOL of patients with PSC, and the PBC-10 questionnaire to evaluate the QOL of patients with PBC. For patients with AIH, no QOL questionnaire was applied, due to the small number (n = 5) of enrolled patients.

General data were collected on patients registered on the platform from 1 April 2023, to 15 July 2025. Fifty-five patients with PBC, 21 patients with PSC, and five patients with AIH were registered on the website.

To evaluate the usefulness of the online platform, a non-standardized questionnaire was administered. It assessed the degree of satisfaction, the quality of the information provided, the influence on lifestyle, the level of pre-existing health literacy, and the improvement after using the online platform. It contains 30 questions about diagnosis, stage of the disease, daily phone use, level of education, demographics, and level of health literacy.

To evaluate if QOL questionnaires on PSC and PBC can be administered online as a pilot study, we used the PBC-10 questionnaire, which was administered to PBC patients, comprising 10 questions. The answers are scored on a Likert scale from 1 to 5, with a higher score indicating a worse QOL. For PSC, the CLDQ-PSC questionnaire was administered, consisting of 24 questions. The answers are rated on a 1 to 7 Likert scale, where a higher score signifies a better QOL. The questionnaires were translated from English into Romanian, maintaining the meaning of each question and answer. Two researchers (M.M. and L.S.G.) with proficiency in both English and Romanian translated the questionnaires. They reached a consensus to identify any differences and ensure the questions retained their clinical relevance, while using language accessible to individuals without medical knowledge.

The results of the questionnaires were divided into the subdomains described earlier [39,44,54]. Therefore, for the CLDQ-PSC questionnaire, we categorized the answers into the following subdomains: Fatigue, Worry, Symptoms, Emotional, and Sleep. For the PBC-10 questionnaire, we categorized the answers into the subdomains: Pruritus, Symptoms, Fatigue, Cognition, and Social.

The answers were converted into percentages based on the answer type and then displayed as histograms. The two QOL questionnaires were evaluated in this pilot study through basic assessment only to determine the overall clinical impact and the reliability between the questions in each questionnaire. We thus conducted a pilot feasibility study, not a validation study for them. The results are presented in Supplementary Materials.

The QOL questionnaires and the evaluation questionnaire were applied using Google Forms. The analysis of the collected data was carried out using Google Sheets (Google LLC, 2025, Mountain View, CA, USA), Microsoft Office Excel (Microsoft Corporation, Version 2021, Redmond, WA, USA) for graphical representation, and DATATab (DATATab Team. DATAtab: Online Statistics Calculator. DATAtab e.U. Graz, Austria. URL https://datatab.net/) for statistical analysis. Descriptive statistics were used to summarize patient demographics and the results of the questionnaires. The comparative analysis was performed using either the Chi-square test or the Wilcoxon test. For the reliability assessment of the platform evaluation questionnaire questions, Cronbach’s alpha was calculated for each field. Significance of comparisons is indicated by *p* values. The significance level was set at *p* < 0.05.

### 2.3. Participation Flow

To register on the platform, individuals must read and agree to the site’s terms and conditions, which are established in accordance with the General Data Protection Regulation (GDPR) to protect personal data. We collaborated with the lawyer of the Fundeni Clinical Institute hospital in Bucharest to ensure compliance with European and Romanian laws, and subsequently obtained approval from the ethics committee. Since its establishment, the platform has been administered by the Romanian Association of Liver Diseases (RoALD). The registration flow and project implementation are presented in Appendix A.

The registration on the site includes the following details: name, contact information, date of birth, type of diagnosis, location of residence, location of the medical center monitoring, attending physician, and date of diagnosis. Registration on the platform is free of charge and non-profit to avoid any advertising that might impact the integrity and perception of users regarding information bias.

Each registration is verified by a physician from the team that coordinates the online platform and then accepted or refused.

Each patient signed up for the online platform after hearing about it from their attending physician or family doctor, discovering it through an online search, or encountering it in presentations at patient conferences and national gastroenterology and hepatology conferences. By accessing the website, each patient can find general information about each disease.

Patients with a valid account on the online platform are eligible if they have been diagnosed with PSC, PBC, or AIH, as confirmed by a doctor involved in the project who has evaluated the patient before or after enrollment. Once confirmed in the platform, the patient can access the discussion forum and receive questionnaires and updates about the disease or events dedicated to patients via email. For administering the questionnaires, links to online forms were sent via email based on the patients’ diagnoses and purposes.

## 3. Results

### 3.1. Content of the Online Platform

The online platform fulfills several roles:

- Educational role, by providing information about the three diseases;
- Community role, through the discussion forum;
- Registry role, gathering of a minimum set of data that can be used for a future medical registry;
- Facilitation of patients’ contact with medical professionals and specialized medical services.

The information contained on the website provides general guidance on diagnosis, epidemiology, monitoring, treatment, nutritional aspects, and physical activity.

The general data on the patients on the website, presented in Table 1 and collected from the platform’s database, showed that females were more common for all three diseases; most patients were from urban areas; and patients with PSC had a lower mean age at diagnosis compared to those with PBC or AIH. The information was obtained by using the data provided by the platform, which is exported in a format for statistical use.

In addition to the information available on the website, patients can access a booklet published in 2024. This booklet presents similar information in both physical and electronic formats, including original illustrative and explanatory images about the two diseases.

**Figure 1:**
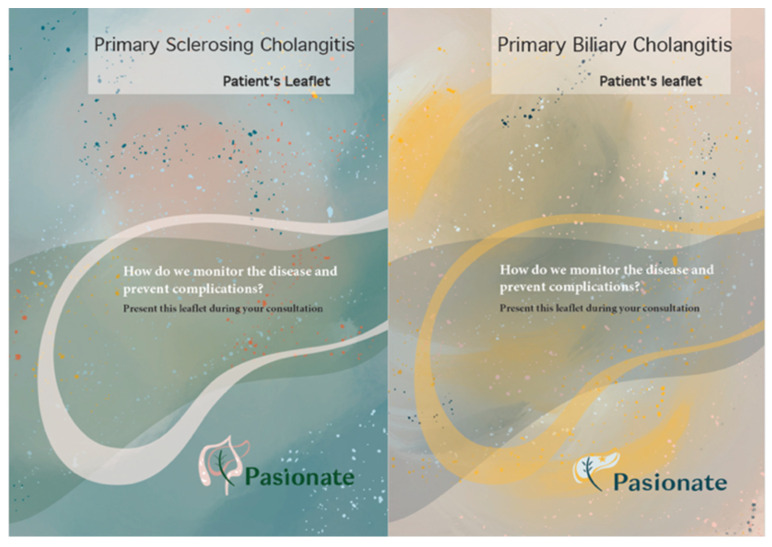
The front page of the booklets given to patients for educational and monitoring purposes. The figure is adapted from the Romanian language version. The original version is available on the online platform (https://www.colangite.ro).

The printed version helps distribute this information to patients during diagnosis, providing them with the initial understanding they need about the disease. The front page of the booklet (translated into English) is shown in Figure 1. Additionally, the booklet serves as a record of the investigations performed and their dates, supporting long-term monitoring.

### 3.2. Utility of the Online Platform

**Figure 2:**
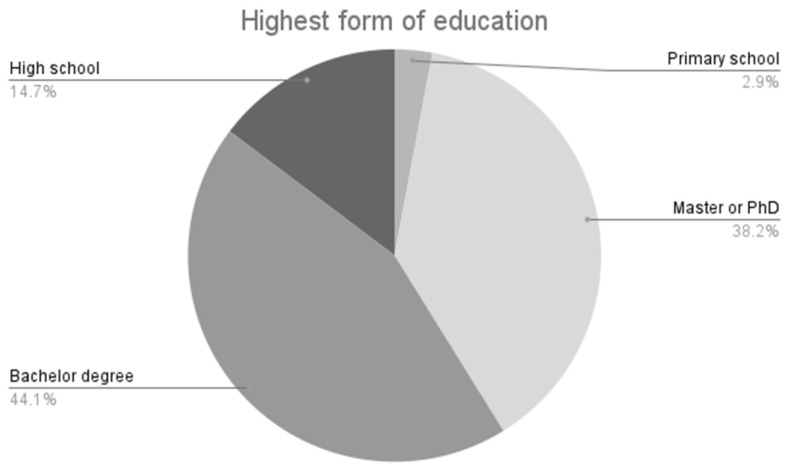
The highest form of education completed by the respondents to the applied questionnaire, 82.3% responded that they had finished university.

One of the questions asked patients through the non-standardized questionnaire was about the type of education they completed. It was found that 82.3% held a bachelor’s degree, a master’s degree, or a PhD. In comparison, only one patient (2.94%) reported having only completed primary school, as shown in Figure 2. The data among the patient categories based on education level were compared using the Wilcoxon test, revealing significant differences (*p* < 0.001). We also conducted a comparative analysis using the Chi-square test with diagnosis (*p* = 0.891), living area (*p* = 0.872), and liver cirrhosis (*p* = 0.667), but no significant differences were found between the groups.

When asked about the importance of medical education, 82.4% of patients considered it a public health problem. Additionally, all respondents believed that medical education should be included in everyone’s education.

**Figure 3:**
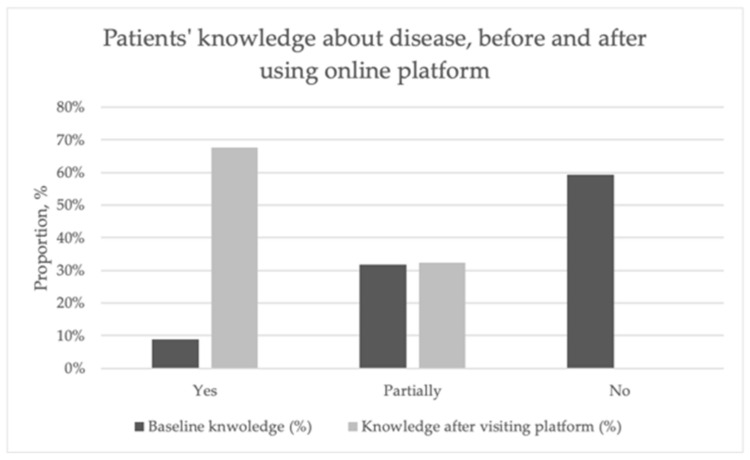
Improvement in patients’ medical knowledge about PSC-PBC before and after using the online platform. The results in this figure comprise the responses to questions before using the platform and those after using the platform. The patients reported improvement in their health literacy about their disease.

Considering that the online platform functions as a patient education tool, bringing information from international guidelines translated into Romanian and non-technical language, we asked questions about how patient health literacy was improved after using the platform and the level of medical knowledge, as shown in Figure 3. In this figure, we composed questions about the disease related to knowledge before accessing the platform about diagnosis, monitoring, prognosis, and treatment. We compared them with the answers to the question about knowledge after accessing the platform.

Thus, we can observe that at the baseline, 59.4% of patients responded with “No” and 31.7% responded with “Partially”. Regarding the question about knowledge improvement after visiting the platform, 67.6% responded “Yes”, and 32.4% answered “Partially”. No patient responded with “No” to the follow-up question. The differences between the answers to the initial questions were compared using the Chi-square test, which were significant (*p* = 0.002). However, when comparing the answers to the question on improving knowledge, no significant differences were observed. We compared the reliability of the questions about the baseline knowledge using Cronbach’s alpha, and we obtained a coefficient of 0.720. When we added the question about improving knowledge, the coefficient was 0.630.

**Figure 4:**
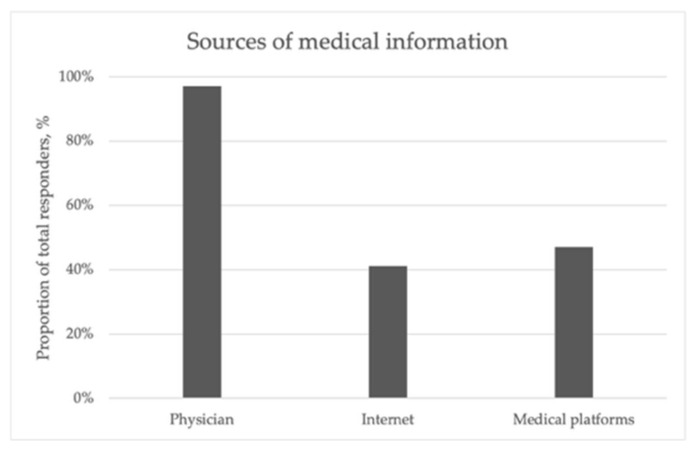
This figure shows the sources of medical education for the patients from the platform. Most of them (97.1%) indicated that their physician (primary care or specialist) was the main source of information.

The online platform can be accessed from both computers and mobile phones or tablets, being optimized for all types of devices. As shown in Figure 4, regarding health literature sources, 97.1% of patients cited their gastroenterologist or primary care physician as their primary source, while 41.2% used online search engines and 47.1% consulted medical websites.

## 4. Discussion

This paper outlines the approach taken to develop Romania’s first online platform dedicated to patients with PSC, PBC, and AIH. The platform aims to include patient registration, sharing of medical information, and a community space where patients can connect with hepatology specialists to ask questions.

Health literacy helps determine when to seek medical support. For patients with PSC or PBC, it is especially challenging due to limited, differing information compared to other chronic diseases. Therefore, our platform aimed to fill this gap by providing medical knowledge and facilitating patient–physician interaction [42].

The achievement of health literacy depends on the general level of education, the ability to use electronic tools, access information, and the age of the patients. Thus, younger patients prefer electronic methods, such as mobile applications and the integration of portable devices. In comparison, older patients prefer printed materials or those in the form of websites or television advertisements [55,56,57].

We aimed to reach patients via an online platform that allows access from multiple devices, including personal computers and mobile phones [51]. We acknowledge that the self-selection of patients with high education levels from urban areas may be a bias regarding the target population. To reach the population that has difficulty accessing online support, we created printed booklets that are also available online to registered patients. In comparison, another similar educational intervention was conducted by van Munster et al., who aimed to assess the patients’ knowledge of PSC using explanatory videos [21].

The implementation of health literacy methods requires overcoming language and comprehension barriers, both from the perspective of medical language and everyday language, but also in the creation of materials translated from international languages (English) into the local language [20,33,55,58]. Another challenge is access to the Internet, mobile devices, and digital literacy, which affects those from disadvantaged backgrounds [56]. Thus, the possibilities of transmitting information can be classified, depending on the medium, into:

- Digital methods—eHealth literacy: where dedicated websites, online platforms, social media pages and groups, mobile applications on phones, tablets, and smartwatches for transmitting information are available [55,56,59]. They can update information, display images and videos, integrate clinical evaluation calculators, and facilitate patient-to-patient or patient-to-physician interaction. The main disadvantage is their content, which can sometimes come from unverified sources [56,60,61]. To address this, our platform is managed by hepatologists, who filter and transform the information to make it easy to understand while maintaining accuracy.
- Classic, non-digital methods: printed materials, flyers, TV or radio commercials, and medical books. They have proven usefulness and contain information material verified by competent authorities; however, the disadvantage is that it is more difficult to keep updated information, given that the field of autoimmune liver diseases receives new data every year [34,35,62,63]. The booklets produced in 2024 contained the most current information, with original images created by a digital designer in collaboration with physicians [1,64].

One aim of this study was to assess the feasibility of administering QoL questionnaires online through the platform, as presented in Supplementary Material S1. Although still in the early stages, these results will enable us to conduct a comparative study using data from both online environments and printed questionnaires, and also explore the possibility of applying questionnaires periodically [65]. One study, conducted by van Munster et al., utilized a mobile app to assess the quality of life of patients with PSC, employing a simple quality of life score. The study aimed to evaluate the feasibility of administering questionnaires in this manner, with results comparable to those obtained through traditional, in-person administration. However, this app was only designed for QoL and was developed in collaboration with a private partner. A similar application can be developed by combining digital health literacy and QoL questionnaires [66,67].

The evaluation of the questionnaires and their usefulness was only initially assessed to determine if they could be applied online, and pilot data were collected. We found that the reliability and consistency data were similar to those in the validation studies, with the CLDQ-PSC study by Younossi et al. reporting Cronbach’s alpha values of 0.85–0.94, and the other study by Alrubaly et al. showing values of 0.91–0.93. These results are promising for future validation of the questionnaires in Romanian and for online application [39,44].

The utility of the platform can also be measured by the level of user satisfaction and compliance with health programs, treatments, and medical monitoring, which is more difficult to quantify [39,68,69]. It is important to measure the level of health literacy to improve the process of informing patients, making it more accessible, efficient, and easier for them to understand [20,34]. The evaluation method used was non-standardized, which makes it difficult to interpret the information obtained on digital literacy levels [36,37,70]. One future consideration is to evaluate the level of digital health literacy over the long term using standardized scales, which will enable precise correlations. For this study, a pre-registered primary outcome was set before starting enrollment of patients from the platform.

The data on health literacy in Romania are limited. One study that used a general questionnaire on general health literacy in rural areas reported a reliability coefficient of 0.809 for the functional literacy scale [19]. Meanwhile, a study assessing digital health literacy among medical students during the COVID-19 pandemic, which utilized a standard tool, reported a reliability coefficient ranging from 0.740 to 0.768, depending on the domains of the questions included. These values are comparable to the pilot study data on patients’ basic medical knowledge, which showed coefficients of 0.720 and 0.630, respectively, when the question on post-use platform evaluation was included [19,71].

Various online platforms, managed by medical professionals and patient organizations, provide patients with PBC or PSC with information about their disease, diagnosis, monitoring, treatment options, and the importance of knowledge and support [60,72]. These platforms enable patient registration, collect minimal legally compliant information, encourage patient communities, facilitate medical service integration, and offer opportunities for clinical trials [60,73]. Notable platforms include the PSC-Partners (US, 2005 [74]), PSC support (UK, 1995 [75]), and ALBI (France, 2007 [76]), which also covers other hepatobiliary diseases, such as PBC and AIH. For PBC patients, the platforms include the PBC Foundation (UK [77,78]) and PBCers (US [78]). European organizations like ERN-Rare Liver and FILFOIE facilitate registration, information, doctor-patient partnerships, and collaborations between researchers [38,72,79,80]. In Romania, the “Colangite.ro” platform is the first initiative of its kind, specializing in and directed towards patients affected by PSC, PBC, and AIH. By creating it, we aimed to foster a doctor–patient partnership that enhances health literacy, treatment adherence, and monitoring, while also promoting patient involvement in clinical trials. This project also serves to provide a replicable model for others to adapt and implement. These medical and patient associations are decentralized at the country or regional level, with multiple centers forming a network for research, investigation, and disease monitoring [13,38]. We have attempted to achieve this by establishing a network of medical centers to manage the diagnosis, monitoring, and treatment of these patients.

The external validity of this study was limited by its small sample size, which included a small number of patients. Patients were not specifically selected; all individuals enrolled in the platform were included, and the participants in the evaluation were not preselected. This is also due to the limited data available in the literature on these diseases in Romania, making their true impact unclear. Consequently, the findings are difficult to generalize or compare with other diseases that may have culture-specific differences [33,37,46]. In our study, a self-selection bias also occurred, as patients volunteered to participate rather than being selected based on their type of pathology. This bias tends to over-represent individuals who are more digitally literate or interested in digital tools, which can skew perceptions of acceptability. This issue is standard; for example, studies on eHealth in cirrhosis acknowledge that sufficient technological literacy (or a knowledgeable caregiver) is essential for engagement [36,68,81].

Our model can be applied to other diseases and is practical in Low- and Middle-Income Countries (LMICs) due to its low implementation costs [52]. Challenges include the digital health divide, which is hindered by barriers to education, access, and infrastructure. Nutbeam et al. revealed that even in Europe, low health literacy exists, suggesting LMIC populations may face greater difficulties with digital health tools amid health disparities [30].

Clinicians who diagnose PSC, PBC, or AIH must learn to communicate with patients in a language adapted to their needs, despite the complexities of these pathologies, which require a higher level of understanding [33,82]. This platform serves as a means of helping physicians transmit information more easily, requiring only registration for the patients on the platform to increase their level of health literacy. The potential information that will be collected through the online platform, regarding the level of health literacy, quality of life, and response to treatment, will be able to help policy stakeholders to make decisions that have an impact on patients. The publication of the results obtained will form the basis for future legislative proposals aimed at attracting research funds and financing treatments [45,52].

This paper had several limitations. The study had a relatively small sample size, despite being included over a 2-year period, which makes it difficult to generalize the data obtained, particularly in exploratory or pilot studies [33,37,83]. Patients were self-selected, as participation was voluntary, attracting participants who were more motivated in finding information about the disease, resulting in bias toward those who registered [81,84]. This selection bias was reflected in the demographic profile of the participants, who tended to have higher education levels, more digital knowledge, and were primarily from urban areas. This demographic skew, evident in online surveys, limits the representativeness of the results for the general population, particularly for underserved and rural populations [83,85]. The strategy to overcome the barriers for underserved populations should involve using telemedicine to address geographical challenges and reduce patient burden [48,86]. This approach must include accessible technologies and interventions developed in collaboration with vulnerable populations [52]. This can involve simplifying language and incorporating helper images. Additionally, working with local community organizations, the local administration, and family doctors can provide technical support and help build trust [52,87].

However, differences were observed between digital knowledge and health literacy. This is important because a higher level of traditional health literacy does not necessarily lead to a better ability to evaluate digital medical information, possibly due to lower skills in understanding digital information [37]. Another limitation involved the evaluation and interpretation of the data. The lack of a standard questionnaire limits the ability to generalize the results. The use of self-reported measures can introduce bias due to personal interpretation and the desire for social approval, thereby reducing reliability [41,84]. Additionally, the computer analysis lacked advanced statistical calculations, making comparisons difficult. This also affected the analysis of the QoL questionnaires, as data could not verify disease status (such as clinical severity scores and laboratory values). Consequently, comparisons were made based on each patient’s self-assessment of the level of impairment, which limited the ability to draw definitive conclusions about the cohort’s actual condition. This approach enables the identification of associations, but establishing causal relationships remains challenging [38,88].

As Romania has the lowest education level among young people in the EU (23.2% in 2024), according to Eurostat, future efforts to improve health literacy should target those with lower education levels, unlike the participants in the current study [89]. This will involve adapting the questionnaire’s accessibility for use on social media platforms, which are the most frequently used. Long-term patient monitoring will be achieved by introducing an application that periodically tracks disease activity and quality of life, with validation through traditional questionnaires during consultations. Standard questionnaires will also be included for patients with AIH, in addition to PSC and PBC. Standard health literacy assessment scales will also be used prospectively, allowing for comparisons with other studies. A longer follow-up will enable the expansion of patient groups, allowing comparisons to be made between urban and rural populations, as well as among the diseases included in the platform. Patient selection bias may persist, as individuals can register themselves on the site; however, each account is verified for quality before approval. Addressing rural populations remains a challenge, as Romania’s healthcare system still faces difficulties in these areas, and information transmission is hindered; however, this could be mitigated through the efforts of primary-care physicians.

## 5. Conclusions

This article outlines the methodology for developing and implementing a digital platform for patients diagnosed with PSC, PBC, or AIH, which is intended to be the first online resource in Romania aimed at improving patient health literacy and fostering effective doctor–patient communication. It discusses some of the challenges involved in providing health literacy, facilitating physician–patient interaction, and utilizing digital devices for administering questionnaires.

The platform has incorporated features such as general guidance for diagnosis, monitoring, and treatment, and promotes community engagement via a discussion forum and a patient registry to connect patients and doctors. A pilot feasibility study was conducted using a non-standardized questionnaire. Additionally, standard QoL questionnaires were employed during the pilot to assess patients and to explore the feasibility of administering these assessments online. Results indicated that fatigue was a common symptom in patients with PSC and PBC, while pruritus was more frequently reported in patients with PBC. The study was subject to a significant self-selection bias, reflecting the characteristics of the target population.

Looking ahead, this platform has the potential to serve as a model for enhancing digital health literacy in other chronic diseases. It might be integrated into clinical practice to facilitate patient monitoring, especially regarding QoL, if a mobile monitoring app is developed. An important future development could involve connecting the platform with a medical registry, enabling researchers conducting clinical trials to communicate more easily with patients. Long-term data collection and expanding the platform to include more patients could support disease impact studies in Romania, which may contribute to the approval of new treatments, funding for health programs focused on diagnosis and monitoring, and improved access to medical services and education for patients.

## Figures and Tables

**Table 1:** Descriptive information about the patients registered on the platform. The data are categorized by disease types (PSC, PBC, and AIH). The table displays basic descriptive information. No formal comparative analysis was performed. Most patients were from urban areas, female, and the age at diagnosis was lower in PSC patients.

	PSC Patients (N = 21)	PBC Patients (N = 55)	AIH Patients (N = 5)
Female sex, %	81.8%	89.09%	100%
Urban/rural area, %	66%/34%	74.5%/25.5%	80%/20%
Age at diagnosis, average (CI95%)	37.71 (31.15–44.27)	47.84 (44.74–50.93)	51 (31.91–64.44)
Variant syndrome, %	13.64%	7.27%	40%
Liver transplant, %	22.7%	16.6%	0

**Figure A1:**
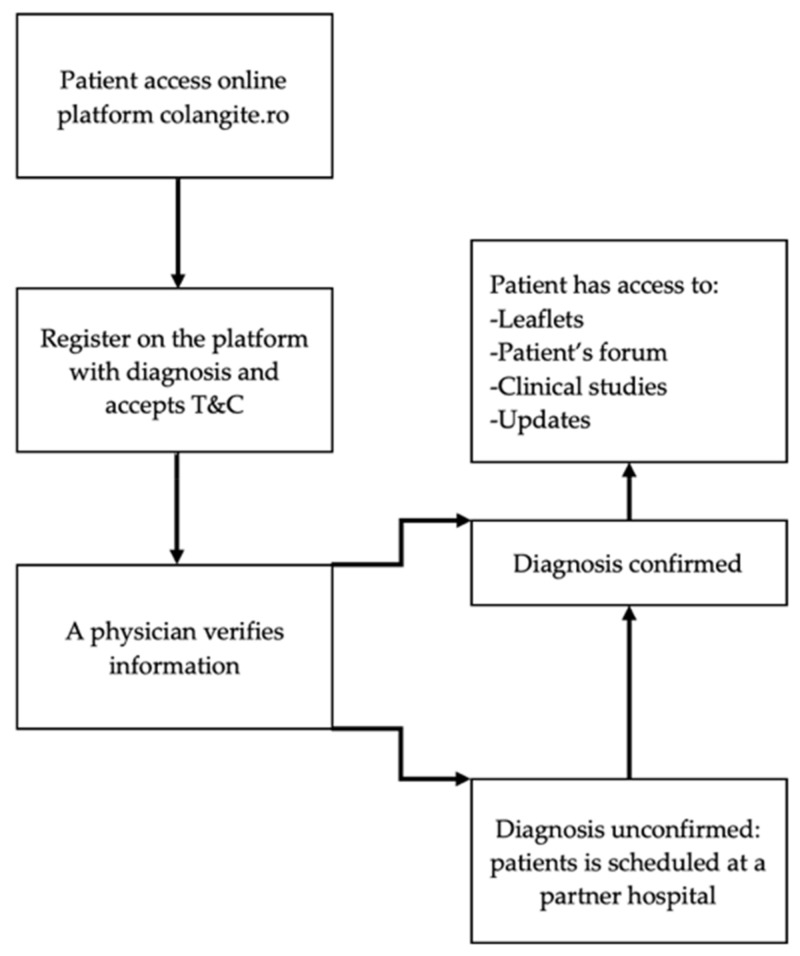
Flow diagram for registration of patients on the platform.

## Data Availability

The data on which this study is based will be made available upon request. Booklets containing information on the diseases are available for patients on the website https://www.colangite.ro.

## Supplementary Materials

The following supporting information can be downloaded at: https://www.mdpi.com/article/10.3390/healthcare13233148/s1, Figure S1. Responses to the CLDQ-PSC questionnaire by domain, represented as histograms. (A) Fatigue; (B) Worry; (C) Symptoms; (D) Emotional; (E) Sleep. Figure S2. Responses to the PBC-10 questionnaire by domain, represented as histograms. (A) Pruritus; (B) Symptoms; (C) Fatigue; (D) Emotional; (E) Sleep. Table S1. Scores distribution and reliability analysis for CLDQ-PSC questionnaire. Table S2. Scores distribution and reliability analysis for the PBC-10 questionnaire.

## Abbreviations

The following abbreviations are used in this manuscript:

PSC	Primary Sclerosing Cholangitis
PBC	Primary Biliary Cholangitis
AIH	Autoimmune Hepatitis
WHO	World Health Organization
mDiHERS	Mobile-Centered Digital Health Readiness—Health Literacy and Equity Scale
PROs	Patient-Reported Outcomes
CLDQ-PSC	Chronic Liver Disease Questionnaire—Primary Sclerosing Cholangitis
PBC-10	Primary Biliary Cholangitis 10-item Questionnaire
PBC-40	Primary Biliary Cholangitis 40-item Questionnaire
GDPR	General Data Protection Regulation
RoALD	Romanian Association of Liver Diseases
EASL	European Association for the Study of the Liver
AASLD	American Association for the Study of Liver Diseases
CI95%	Confidence Interval 95%
PSC-PRO	Primary Sclerosing Cholangitis Patient-Reported Outcome
UK	United Kingdom
ALBI	Association of Patients with Rare Hepato-biliary Diseases
US	United States
ERN	European Reference Network
FILFOIE	Filière des maladies rares du foie
TV	Television
CCM	Chronic Care Model
HBM	Health Belief Model
PPM	PRECEDE-PROCEED model
eHEALS	eHealth Literacy Scale
SCT	Social Cognitive Theory
QOL	Quality of Life

## Appendix A

Several elements were needed to implement the project:

- Team involved:
  ○ Hepatologists, who know the necessary information about the disease and write the information material for patients;
  ○ Patient association, which knows the needs of patients and can help translate informational materials, relate to patients, and adapt language;
  ○ Digital designer, the person who works with others (physicians, patients’ representatives) to create informational materials and develop the online platform.
- IT and stationery equipment:
  ○ The main computer on which the online platform is built, where the offline database containing the informative materials is stored, and the informational analysis is produced;
  ○ Web-hosting: required for a web address;
  ○ Website creation and administration platform;
  ○ The company that publishes and prints the informational materials, leaflets, and promotional posters.
- Informative materials

The flow for registering and enrolling patients in studies, via the online platform, was described in the Materials and Methods section. Figure A1 shows the registration flow diagram.
