# Supplementary material for: From Isolation to Information: Launching an Online Community for Patients with Primary Sclerosing Cholangitis, Primary Biliary Cholangitis, and Autoimmune Hepatitis in Romania—A Descriptive Study
Source: Healthcare (Basel). 2025 Dec 2;13(23):3148. doi: 10.3390/healthcare13233148 (PMC12692238; doi:10.3390/healthcare13233148)
Supplement: Supplementary file 1 [file healthcare-13-03148-s001.zip › healthcare-3946198-supplementary.pdf]

Supplementary Materials

Feasibility evaluation pilot for quality of life questionnaires

The questionnaires had 25 respondents for PBC and 11 for PSC, and 34 for the general non-standardized questionnaire. The questionnaires were sent individually and anonymously to the patients, without being analyzed against their known medical data. Therefore, the aim was not to validate the questionnaires. The questionnaires were administered from March 3, 2025, to April 19, 2025.

The assessment of the QOL using the PBC-10 questionnaire and the CLDQ-PSC questionnaire is presented in Supplemental Figures S1 and S2. Since the aim was to determine whether questionnaires could be administered online and to collect patient information about the disease's impact through an online platform, this pilot study did not gather data to identify patients for comparison with the medical data available at the centers where they are evaluated, to validate the questionnaires.

In Figure S1, regarding patients with PSC, responses grouped into domains show that symptoms related to the disease (pain, pruritus, abdominal discomfort) are the least significant, with 59.1% of patients answering 1 or 2. Fatigue is reported to have a moderate impact, with 47% of patients responding with a score of 3 or 4. Responses concerning worry are the most heterogeneous, displaying the least skewness, while sleep is affected in 40.9% of patients (responses 1, 2, or 3).

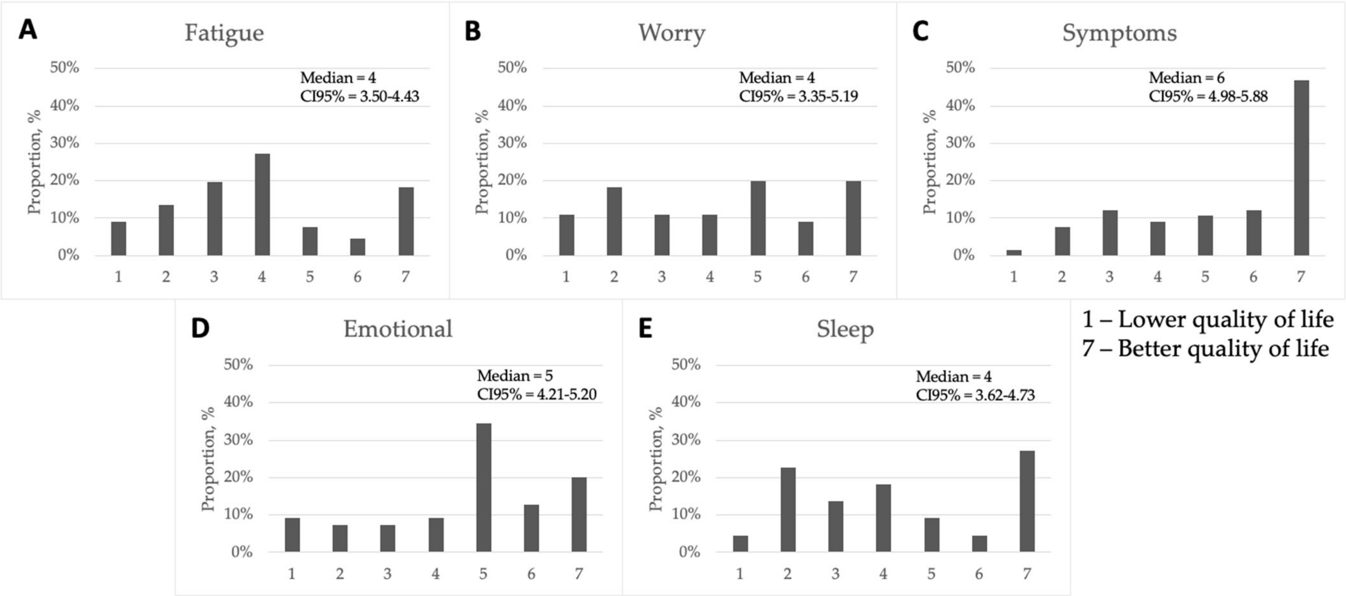

.

Supplemental Figure S1. Responses to the CLDQ-PSC questionnaire by domain, represented as histograms. (A) Fatigue; (B) Worry; (C) Symptoms; (D) Emotional; (E) Sleep.

Supplemental Table S1. Scores distribution and reliability analysis for CLDQ-PSC questionnaire

Scale	Mean	SD	Cronbach's alpha
**Fatigue**	3.06	1.79	0.96
**Worry**	4.27	2.02	0.94
**Symptoms**	5.43	1.81	0.92

**Emotional**	4.70	1.82	0.96
**Sleep**	4.18	2.03	0.92
**Overall**	4.56	1.97	0.98

In Supplemental Table S1, we present the descriptive data for the questions included in the CLDQ-PSC questionnaire, based on the previously defined areas of applicability. Values above 0.90 are observed for Cronbach's alpha coefficient, both overall and within each domain.

In Supplemental Table S2, we presented the descriptive and reliability data for the applications of the PBC-10 questionnaire. We observed a calculated Cronbach's alpha coefficient of 0.94, with values of 0.88 for questions on symptomatology and 0.83 for the social domains. Pruritus, fatigue, and cognition each have only one question; therefore, no reliability analysis was performed.

Supplemental Table S2. Scores distribution and reliability analysis for the PBC-10 questionnaire

Scale	Mean	SD	Cronbach's alpha
**Pruritus**	2.2	1.62	-
**Symptoms**	2.96	1.20	0.88
**Fatigue**	3.04	1.24	-
**Cognition**	2.56	1.16	-
**Social**	2.68	1.28	0.83
**Overall**	2.76	1.28	0.94

In Supplemental Figure S2, regarding patients with PBC, shows that pruritus has a small impact on QoL (44% of patients responded 1 or 2), the impact on social life (53.3%), and the impact of symptoms (55%) was reported as moderate (response 3 or 4), while fatigue (64%) was the most important factor reported (responses 3, 4 or 5).

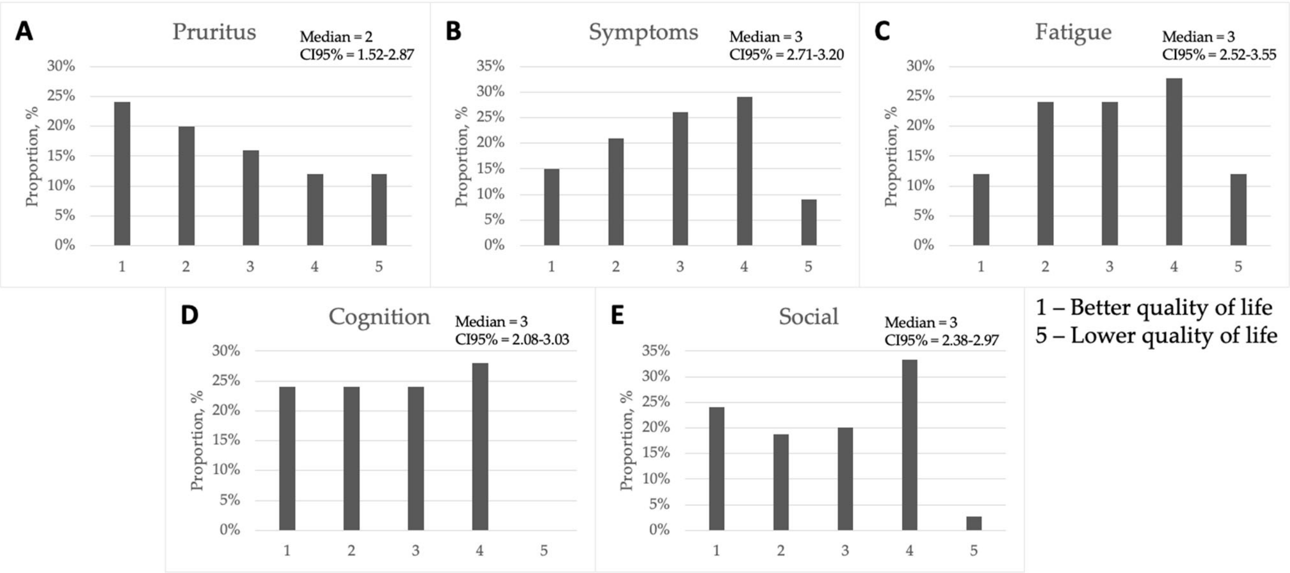

Supplemental Figure S2. Responses to the PBC-10 questionnaire by domain, represented as histograms. (A) Pruritus ; (B) Symptoms; (C) Fatigue ; (D) Emotional; (E) Sleep.
